# Strain Rate Sensitivity of Polycarbonate and Thermoplastic Polyurethane for Various 3D Printing Temperatures and Layer Heights

**DOI:** 10.3390/polym13162752

**Published:** 2021-08-17

**Authors:** Nectarios Vidakis, Markos Petousis, Apostolos Korlos, Emmanouil Velidakis, Nikolaos Mountakis, Chrisa Charou, Adrian Myftari

**Affiliations:** 1 Mechanical Engineering Department, Hellenic Mediterranean University, 71410 Heraklion, Greece; vidakis@hmu.gr (N.V.); mvelidakis@hmu.gr (E.V.); mh90@edu.hmu.gr (N.M.); tm6592@edu.hmu.gr (C.C.); tm6389@edu.hmu.gr (A.M.); 2 Department of Industrial Engineering and Management, International Hellenic University, 14th km Thessaloniki—N. Moudania, Thermi, 57001 Thessaloniki, Greece; apkorlos@ihu.gr

**Keywords:** additive manufacturing (AM), three-dimensional (3D) printing, fused filament fabrication (FFF), strain rate sensitivity, tensile properties, polycarbonate (PC), thermoplastic polyurethane (TPU), nozzle temperature, layer thickness

## Abstract

In this work, strain rate sensitivity was studied for 3D-printed polycarbonate (PC) and thermoplastic polyurethane (TPU) materials. Specimens were fabricated through fused filament fabrication (FFF) additive manufacturing (AM) technology and were tested at various strain rates. The effects of two FFF process parameters, i.e., nozzle temperature and layer thickness, were also investigated. A wide analysis for the tensile strength (MPa), the tensile modulus of elasticity (MPa), the toughness (MJ/m^3^) and the strain rate sensitivity index ‘*m*’ was conducted. Additionally, a morphological analysis was conducted using scanning electron microscopy (SEM) on the side and the fracture area of the specimens. Results from the different strain rates for each material were analyzed, in conjunction with the two FFF parameters tested, to determine their effect on the mechanical response of the two materials. PC and TPU materials exhibited similarities regarding their temperature response at different strain rates, while differences in layer height emerged regarding the appropriate choice for the FFF process. Overall, strain rate had a significant effect on the mechanical response of both materials.

## 1. Introduction

Additive manufacturing (AM) has been one of the most foremost manufacturing technologies over the last 10 years, either in the industrial [1] or in the academic communities [2]. One of the popular AM technologies is fused filament fabrication (FFF), which belongs to them material extrusion category [3,4] of the 3D printing family of technologies and has been widely used for prototyping applications [5]. The increasing demand for the utilization of the FFF technology in various types of applications has significantly raised the interest in the mechanical properties of the polymers, which are manufactured with 3D printing processes [6]. Sufficient research has been conducted on polymer mechanical [7], thermal [8] and other properties [9,10]. However, AM technologies and, especially, the FFF process significantly affect the mechanical behavior of the 3D printed part [10]. This is mainly caused due to the principle of the process. The building material is originally in filament form, which is heated to temperatures close to its melting point and extruded, to be deposited in different patterns in a sequential layer-by-layer fashion, through a nozzle moving in the X and Y directions, until the required geometry is built. This process creates an anisotropic behavior in the built parts [11]. The FFF 3D printing building parameters, such as the layer height, the extrusion temperature and the build pattern, significantly affect the mechanical response of the FFF built parts and have been thoroughly studied in literature [12]. Moreover, the sensitivity of the polymer mechanical properties to flow rate, 3D printing speed and other FFF technology parameters, which are described in more detail below, enhance the anisotropy of the 3D printed parts [13].

AM processed polymers have been thoroughly studied for many different properties in the literature [14,15,16,17,18,19,20,21], while they have also showed promising results for circular economy applications and use [22,23]. Additionally, due to the increasing interest for the use of AM in various applications, extensive research has been conducted to enhance the antibacterial, thermal, electrical and mechanical properties of AM polymers, with the addition of fillers and nanofillers [24,25,26,27,28,29,30]. AM technologies provide the ability to manufacture parts with increased complexity in their geometry, when compared to traditional polymers manufacturing technologies, i.e., injection molding, blown films etc. Although more complex geometries can be manufactured with the utilization of AM technologies, the mechanical behavior of such parts should be carefully studied before their use in applications.

Strain rate is a parameter related with dynamic loading phenomena, which are common in industrial applications [31,32]. Strain rate sensitivity is a measure that has been widely studied for polymers processed through many manufacturing technologies [33]. On the other hand, not enough research exists in the literature regarding the strain rate sensitivity of polymers processed through AM technologies and especially the FFF technology [33,34]. Strain rate sensitivity is an important parameter for end-use applications when polymers are FFF processed. Studying the behavior change due to the strain rate could provide information for fail-safe mechanisms and other dynamic analysis properties [35], such as the energy absorbed in crashes etc. [36].

A recent study provided critical information for the strain rate sensitivity of several different polymers processed with the FFF technology [33]. The increasing demand for FFF manufactured parts with engineering grade polymers such as polycarbonate (PC) and thermoplastic polyurethane (TPU) has further increased the necessity for a study on the strain rate sensitivity of these polymers, which has not been reported before in literature. PC is a well-known high mechanical performance polymer [37], widely used in many applications which require strong dynamical properties, such as automotive [38], medical [39,40,41,42] and optics [43] applications. In AM, PC applications are still limited in the literature, with research mainly related to acoustic applications [44] and heat pipes [45]. TPU is also an elastic material, employed in many industrial and other environmental parts [46,47]. Indicative applications of TPU material are in mechanical seals [48,49] or more advanced applications, such as flexible electronics [50], force sensors [51], pneumatic actuators [52] and pharmaceutical applications [53]. TPU thermal properties in 3D printing have also been reported [54,55].

The strain rate of these materials has been studied either for homopolymers or for composites [38,56,57], but not for parts built with AM technology. The manufacturing of parts through FFF technology requires the extrusion of melted-state polymer in shells, infills and, of course, in consecutive layers [10]. The interlayer and throughout layer fusion are prone to a wide range of process settings, such as number of shells, layer height, infill percentage, infill geometry pattern, extrusion temperature, extrusion speed etc. [58,59]. As mentioned above, AM built parts have an anisotropic behavior. These parameters are among others, responsible for the anisotropic behavior of the parts manufactured with the FFF process [19]. This anisotropy can create unpredicted behavior, especially when dynamic stresses are applied to AM processed parts.

**Figure 1:**
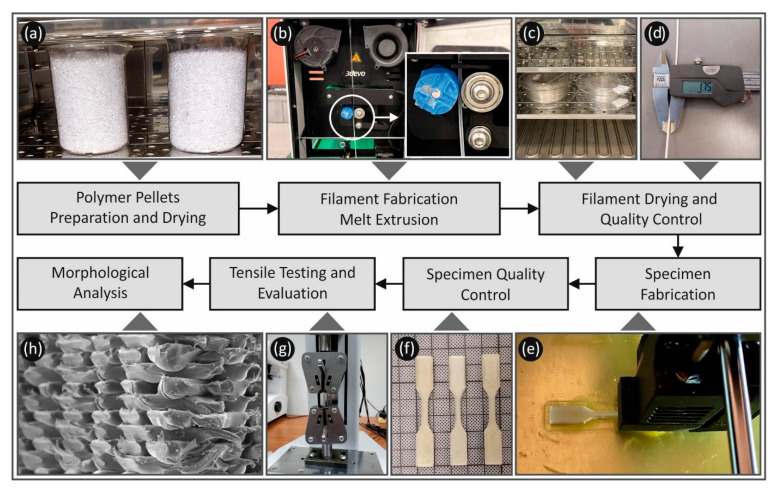
Schematic presentation of the process followed in this work from the specimens preparation to their tests and characterization. (**a**) Pellets drying process, (**b**) filament extrusion process, (**c**) filament drying process, (**d**) filament quality control process, (**e**) specimens 3D printing process, (**f**) tensile test specimens, (**g**) tensile testing of specimens and (**h**) morphological characterization of the tensile test specimens fracture area in SEM.

In this study, PC and TPU were studied for their strain rate sensitivity in five different strain rates, for specimens manufactured with three different layer heights and three different extrusion temperatures. Layer height and temperature have been selected for the strain rate sensitivity, as they are the main 3D printing parameters effecting the inter-layer and intra-layer fusion of the AM processed parts. Strain rates of low shear forces were selected in the study, as such strain rates are mostly developed in applications where FFF parts are introduced. Experimental results were analyzed to thoroughly evaluate the effect of the two 3D printing parameters on the strain rate sensitivity for both materials studied. Calculations of the strain rate sensitivity index ‘*m*’ and toughness were utilized to evaluate the two polymers performance at the different elongation speeds tested herein. Figure 1 summarizes the process followed in the present study. It was found that the layer thickness had a higher effect on the mechanical response of the specimens than temperature and, as expected, the strain rate had a significant effect on the mechanical response for both materials studied, with a similar pattern observed in all cases studied.

## 2. Materials and Methods

### 2.1. Materials

For the purposes of present study, two engineering grade materials were chosen. Polycarbonate (PC), specifically, EMERGE PC 8430-15, was procured from Styron Europe GmbH (Horgen, Switzerland) and thermoplastic polyurethane (TPU) was procured from Ravago Petrokimya Satis VE (Instanbul, Turkey). Ravathane 140 D70 was the specific grade of TPU used. Both materials were in small pellets form and in Table 1 below their properties are shown according to their manufacturers’ technical datasheet.

### 2.2. Filament Fabrication

The filament was produced utilizing a single screw extruder. Prior to the extrusion process, materials were dried for 24 h at 80 °C using an open-loop laboratory oven. The extruder utilized was a 3D Evo Composer 450 (3D Evo B.V., Utrecht, the Netherlands). The filament produced through this process was of 1.75 mm in diameter, with a measured standard deviation of 0.07 mm, controlled through the built-in optical sensor of the device. The extruder barrel consisted of 4 heating zones, with number 1 being closer to the extruder nozzle and number 4 being closer to the hopper. Screw rotational speed (ranging from 2.5 to 15 rpm), cooling fans and winder rotational speed are also parameters that can be controlled either manually or automatically (where applicable). All parameters were experimentally optimized prior to the production of the filament for the present study and the parameters used for both materials are shown to Table 2. The diameter of the filament produced was also statistically measured with a high-quality caliper. Parts of the filament were also tensile tested, to verify the consistency of the produced filament mechanical properties with the material used for each case, prior to its use for the 3D printing of the specimens. In all cases studied no significant deviations were observed.

### 2.3. Fused Filament Fabrication

**Figure 2:**
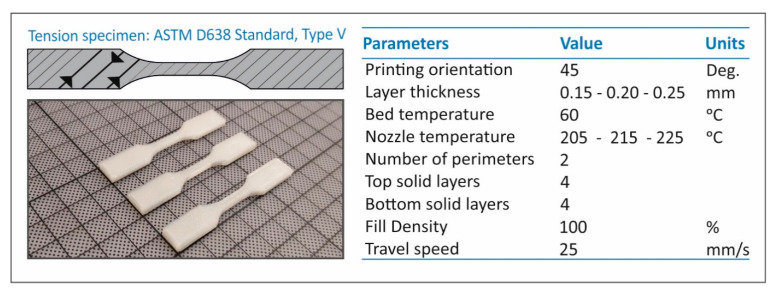
Fused filament fabrication process main parameters set for the manufacturing of the specimens with the tested materials.

FFF AM technology was selected for the purposes of the present study. An Intamsys Funmat HT (Intamsys Technology Co. Ltd., Shanghai, China) FFF 3D printer was utilized for specimen fabrication. Figure 2 shows the 3D printing parameters utilized for the present study. The nozzle temperatures shown in Figure 2 refer to TPU and, apart from that parameter, all other parameters were the same for both materials studied. Corresponding nozzle temperatures employed in the study for the PC polymer were 255 °C, 260 °C and 270 °C. Infill pattern was set to 45°, with a consecutive direction change of 180° at each layer. Additionally, for the PC 3D printing process, the bed temperature was set at 85 °C and the chamber temperature needed to be set up at 65 °C. All filaments were further dried for 4 h at 50 °C before the 3D printing process.

### 2.4. Tensile Stress and Morphological Analysis

Tensile tests were conducted on an Imada MX2 (Imada Inc., Northbrook, IL, USA) tensile test apparatus. Five different elongation speeds ranging from 10 mm/min to 300 mm/min were selected for the investigation of the strain rate sensitivity on the two polymers studied in this work. More specifically, 10 mm/min, 50 mm/min, 100 mm/min, 200 mm/min and 300 mm/min were the tensile test speeds selected in this study. Specimens were manufactured with three different 3D printing parameters regarding the nozzle temperature and the layer height, and all manufactured specimens were tested with the five different elongation speeds. Apart from the elongation speed, all other tensile test specifications were according to the ASTM D638-2 international standard. Type V specimens with a 3.2 mm thickness were fabricated in all cases studied. Tests were conducted at a room temperature of 23 °C.

The side and the fractured area of the specimens were morphologically analyzed through scanning electron microscopy (SEM) on a JEOL JSM 6362LV (Jeol Ltd., Norwood, Massachusetts, United States) electron microscope in high-vacuum mode at a 20 kV acceleration voltage. Prior to SEM investigations, samples were sputter-coated with gold (Au) to avoid charging effects.

## 3. Results

### 3.1. Tensile Properties

**Figure 3:**
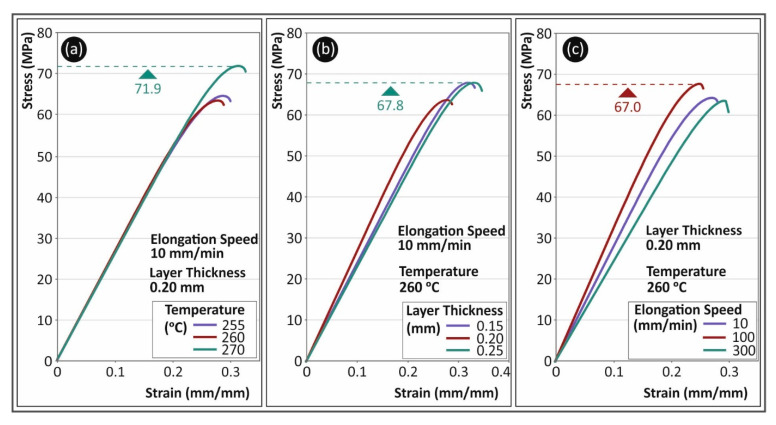
Stress (MPa) to strain (mm/mm) typical curves for PC material for (**a**) different 3D printing temperatures, (**b**) different layer thicknesses and (**c**) different elongation speeds.

Figure 3 shows representative graphs from the tensile tests conducted during the present study. Specifically, in Figure 3a, typical stress (MPa) to strain (mm/mm) curves are shown for PC material for the 10 mm/min strain rate. The layer thickness of the specimens, in this case, was 0.2 mm and curves shown are for the three different 3D printing temperatures studied in this work (one graph per temperature). Through this comparison, it can be observed that increasing the 3D printing temperature to 270 °C improved the mechanical performance of the material, probably due to increased inter and intra layer fusion. In Figure 3b, PC stress (MPa) to strain (mm/mm) typical curves for the different layer thicknesses studied are shown for the elongation speed of 10 mm/min and 3D printing temperature of 260 °C. The figure shows that varying the layer height had little effect on the strength of the specimen, as the difference was less than 10%. This difference could be plausibly attributed to the anisotropy of the process followed (AM). Figure 3c shows the effect of the strain rate on the PC polymer, at three different elongation speeds (10 mm/min, 100 mm/min and 300 mm/min), for specimens built with a 0.20 mm layer thickness at 260 °C. As it is shown, the strain rate had an effect on the typical stress to strain curve of the PC polymer, although the maximum strength developed on the specimens was similar in the three cases shown.

**Figure 4:**
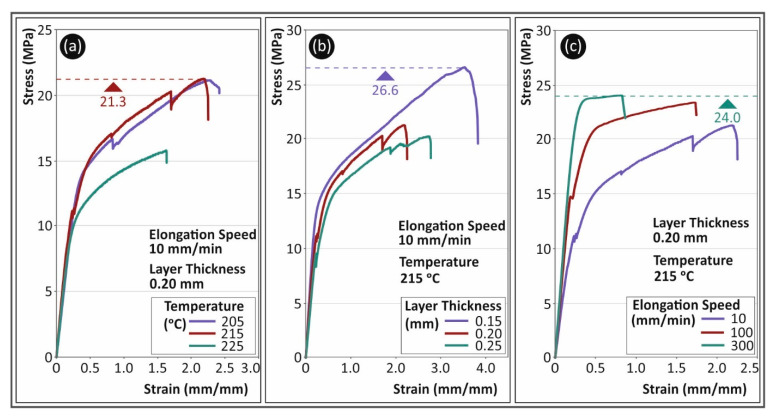
Stress (MPa) to strain (mm/mm) typical curves for TPU material for (**a**) different 3D printing temperatures, (**b**) different layer thicknesses and (**c**) different elongation speeds.

In Figure 4, the corresponding results are shown for the TPU polymer. In Figure 4a, a strong effect of temperature on the behavior of the materials is observed. Results shown could lead to the selection of the optimum 3D printing temperature among the three temperatures studied in this work. The temperature increase had an opposite effect on the TPU polymer, when compared to the PC polymer. When the temperature increased from 215 °C to 225 °C, a decrease of approximately 40% in the tensile strength was observed. It should be mentioned that these sharp drops shown by the stress graphs are caused by local breaks on the specimen, which occurred, during the test, either in its shell or in the infill structure. At higher 3D printing temperatures, TPU material developed smoother curves during the tensile tests. This could be probably attributed to a more isotropic behavior in this case. A better inter and intra layer bonding was possibly achieved, which had a clear effect on the properties of the material, as the specimen became stiffer.

The effect of layer height on the tensile stress behavior is shown in Figure 4b. In this figure, typical stress (MPa) to strain (mm/mm) curves of the TPU specimens tested at 10 mm/min strain rate, with a 215 °C nozzle temperature are shown for the three different layer heights studied. Layer height exhibited a strong effect on the deformation (especially in the non-elastic area) and the tensile strength of TPU material, with lower layer heights developing higher tensile strength and deformation values. The 0.15 mm layer height developed approximately 30% higher tensile strength and almost 25% higher strain values than the 0.20 mm layer height. Figure 4c shows indicative graphs for the TPU specimens from tensile tests with three different strain rate values. Specimens in these three cases were 3D printed with a 0.2 mm layer thickness at a temperature of 215 °C. The strain rate had a strong effect on the brittleness of the material. The increase in the strain rate significantly increased the stiffness of TPU material. The tensile strength, on the other hand, was not affected in the same way, as the difference among the developed values for the three different strain rates was not higher than 10%.

**Figure 5:**
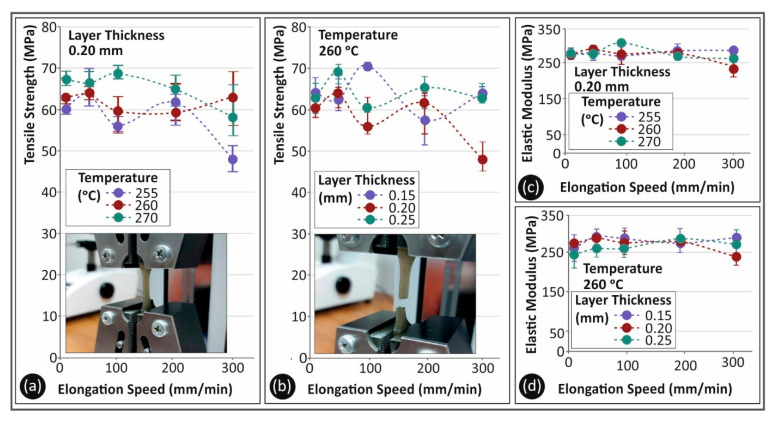
(**a**,**b**) Average tensile strength (MPa) and (**c**,**d**) average tensile elastic modulus (MPa) to elongation speeds (mm/min) for PC material as follows: (**a**,**c**) 3D printing temperatures; (**b**,**d**) layer heights.

Figure 5 shows a comparison between the parameters studied in this work. Specifically, Figure 5a shows, for PC material, the average tensile strength values and their deviation for the different strain rates and 3D printing temperatures studied in this work, for a 0.2 mm layer height. The nozzle temperature created a different trend regarding the tensile strength for the various strain rate values. This was probably due to the different inter and intra layer fusion of the material caused by the different 3D printing nozzle temperatures. The lower sensitivity of the strain rate to the tensile strength was observed for the highest nozzle temperature studied in this work. A similar trend was observed in PC material regarding the sensitivity of the material and the strain rate, when altering the layer height. In Figure 5b, it is shown that the tensile strength for the lowest layer height had a more consistent behavior at the different elongation speeds, when compared to the calculated tensile strengths for higher layer thicknesses. The calculated tensile modulus of elasticity (MPa) is shown in Figure 5c for the different 3D printing temperatures studied and, in Figure 5d, the corresponding values are presented for the different layer heights, for the five elongation speeds tested herein. It can be further assumed that 3D printing temperature constitutes an important parameter affecting the tensile properties of the material.

**Figure 6:**
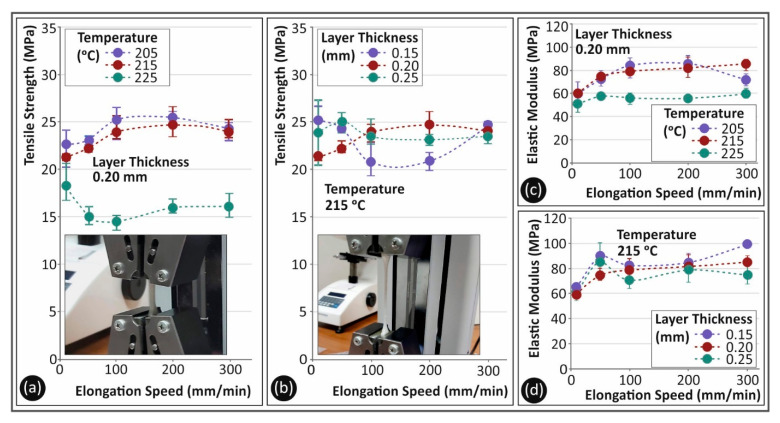
(**a**,**b**) Average tensile strength (MPa) and (**c**,**d**) average tensile elastic modulus (MPa) to elongation speeds (mm/min) for TPU material as follows: (**a**,**c**) 3D printing temperatures; (**b**,**d**) layer heights.

Figure 6 shows the corresponding results for TPU material. It is shown that, for TPU, the nozzle temperature (Figure 6a) had a strong effect on the tensile strength. For the temperatures of 205 °C and 215 °C, low differences were observed regarding the tensile strength, but, when the nozzle temperature was raised to 225 °C, the tensile strength of the material decreased by about 35%. On the contrary, layer height tended to create a different effect on the tensile strength measurements. Higher layer height values were less prone to deviations with the increase in the elongation speed. This could have been plausibly caused by the flow sensitivity of the material and the 3D printer used in the present study.

**Figure 7:**
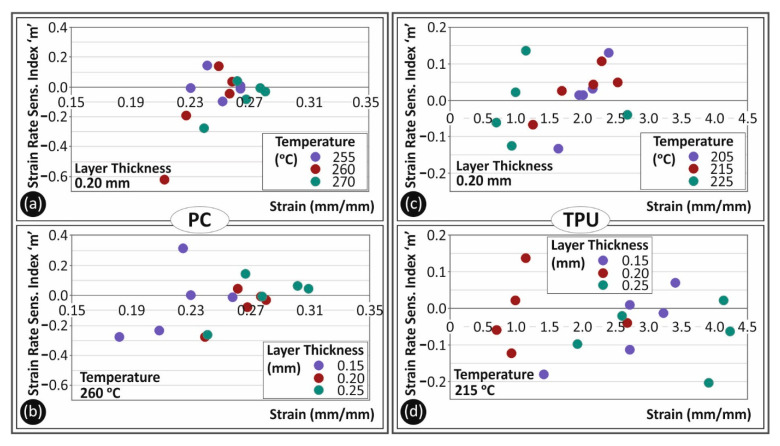
Calculated strain rate sensitivity index ‘*m*’ to strain at break (mm/mm) for PC (**a**,**b**) and TPU (**c**,**d**) materials in comparison with the different temperatures (**a**,**c**) and layer heights (**b**,**d**) studied in this work.

In Figure 7, the strain rate sensitivity index ‘*m*’ is shown in comparison to the 3D printing parameters studied in this work. The strain rate sensitivity index ‘*m*’ is calculated using Equation (1):

(1) $$m=\frac{\Delta ln\left(\sigma \right)}{\Delta ln\left(\dot{\varepsilon }\right)}\;$$

Figure 7a,b refer to PC material, while Figure 7c,d depict the corresponding results for TPU material. Specifically, in Figure 7a,c, the calculated ‘**m**’ index is depicted for the different 3D printing temperatures studied herein for PC and TPU materials, respectively, for specimens built with a 0.20 mm layer thickness. PC tended to create a denser area in the graph, which apparently drives to the conclusion that it is a material less sensitive to low shear force. On the other hand, the 3D printing temperature was an important parameter affecting the tensile properties of TPU material. Increasing the nozzle temperature may cause serious changes to the tensile behavior of TPU material. In Figure 7b,d, the calculated strain rate sensitivity index ‘*m*’ values for the different layer heights studied are shown for PC and TPU materials, respectively, for specimens built with the nominal 3D printing temperature of each material. Although PC tended to be less sensitive regarding the effect of layer height on its tensile strength with the increase in the strain rate than TPU material, layer height affected the tensile behavior of both materials when the strain rate increased.

**Figure 8:**
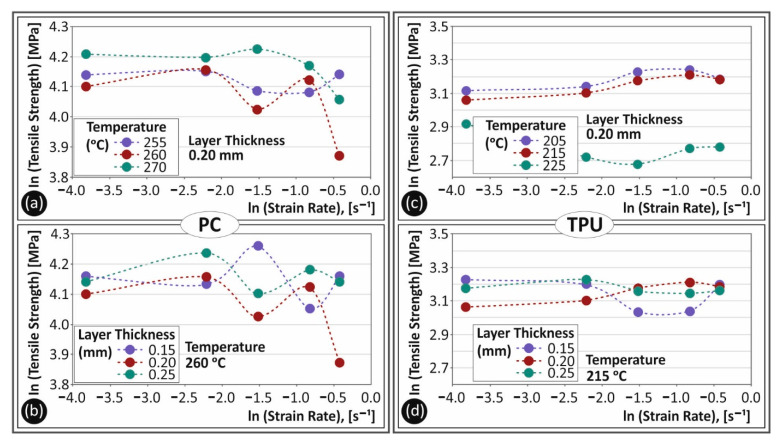
Ln calculated tensile strength (MPa) to strain rate (s^−1^) for PC (**a**,**b**) and TPU (**c**,**d**) materials for the different temperatures (**a**,**c**) and the different layer heights (**b**,**d**) studied in this work.

In Figure 8, the ln calculated tensile strength (MPa) values to strain rate (s^−1^) for the two 3D printing parameters and both materials studied are shown. Comparing Figure 8a,b, the tensile strength differences for the different parameters studied for PC material are depicted. The increase in the extrusion temperature in the 3D printer affected the sensitivity of the material to the strain rate. At a 270 °C extrusion temperature, the material was less prone to tensile strength changes with the increase in the strain rate. Layer height had a similar effect on the strain rate sensitivity of the material, which means that PC had a clearly sensitive behavior to the strain rate for the 3D printing parameters tested. In Figure 8c,d, the respective ln values for TPU material are shown. In these figures, the lower proneness to change of the tensile properties of TPU material can be observed, with the increase in the strain rate, for both the extrusion temperature and the layer height parameters. Nevertheless, it should be mentioned that increasing the extrusion temperature may cause a crucial change in the tensile behavior of TPU material, as it is shown in Figure 8c.

**Figure 9:**
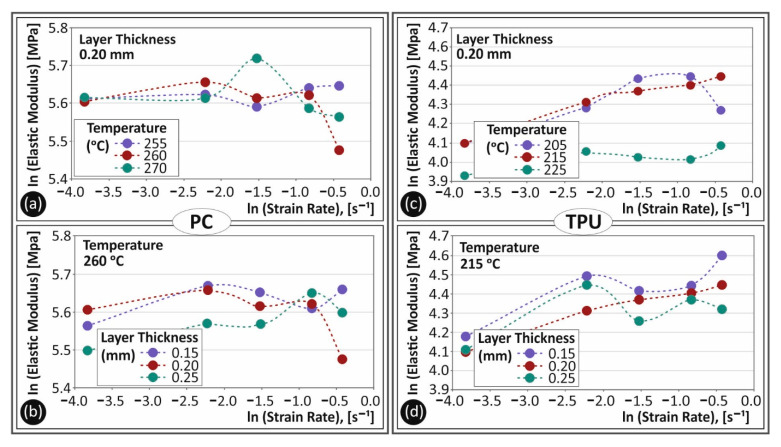
Ln calculated tensile elastic modulus (MPa) to strain rate (s^−1^) for PC (**a**,**b**) and TPU (**c**,**d**) materials for the different temperatures (**a**,**c**) and the different layer heights (**b**,**d**) studied in this work.

In Figure 9, the ln calculated values of the tensile modulus of elasticity (MPa) to strain rate (s^−1^) are shown for both PC and TPU materials. A comparison between Figure 9a,c exhibits the different behavior to temperature and strain rate changes regarding the elastic modulus of the two materials. PC had a rather non-changing behavior, while TPU exhibited a rather intense change with the increase in the strain rate. A different trend was observed for the specimens built with a 225 °C temperature and this was probably due to the change in the behavior of TPU material at this temperature. Figure 9b,d show the trend of the tensile modulus of elasticity to strain rate for the different layer heights studied for PC and TPU materials, respectively. As it is shown, layer height had a lower effect on the strain rate sensitivity of both materials. Differences among all tested layer heights were not significant enough to assume an increased contribution of this parameter to the tensile behavior differences.

**Figure 10:**
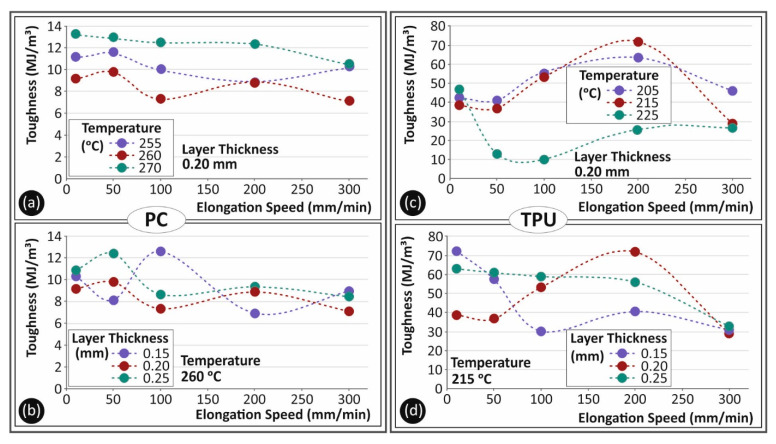
Toughness (MJ/m^3^) to elongation speeds tested (mm/min) for PC (**a**,**b**) and TPU (**c**,**d**) materials for the different temperatures (**a**,**c**) and the different layer heights (**b**,**d**) studied in this work.

In Figure 10, toughness (MJ/m^3^) to elongation speed (mm/min) graphs for the different layer heights and temperatures for both materials are shown. Toughness is the absorbed energy during the tensile test until the break of the specimen. As this parameter considers the plastic deformation area of the stress to strain graph, it is a measure which can be used to describe a ‘fail-safe’ mechanism. As for the strain rate effect on the toughness of PC material, as shown in Figure 10a,b, the strain rate had little effect on the absorbed energy. The temperature increase, shown in Figure 10a, created a better bonded specimen, able to absorb significantly higher energy during deformation. Layer thickness, which is shown in Figure 10b, had little effect on the toughness parameter for PC material for all strain rates tested. In Figure 10c,d, the respective toughness values for TPU material are shown for all the parameters studied in this work. The temperature effect on TPU toughness was far lower than that of PC material. An exemption still existed for the 225 °C temperature, which was already mentioned. Toughness showed to be more prone to layer height change for TPU. It was already shown that a 0.25 mm layer thickness had better tensile behavior. Toughness followed this trend, as the 0.25 mm layer height value was found to create a rather more consistent behavior for all tested elongation speeds.

### 3.2. Morphological Analysis

**Figure 11:**
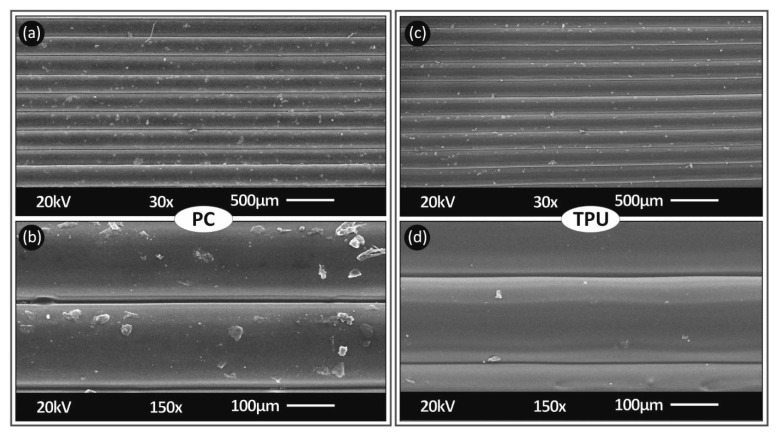
Side surface SEM captures of 3D printed specimens for PC (built at a 260 °C nozzle temperature, with a 0.2 mm layer thickness) (**a**,**b**) and TPU (built at a 215 °C nozzle temperature, with a 0.2 mm layer thickness) (**c**,**d**) materials at 30× (**a**,**c**) and 150× magnifications (**b**,**d**).

**Figure 12:**
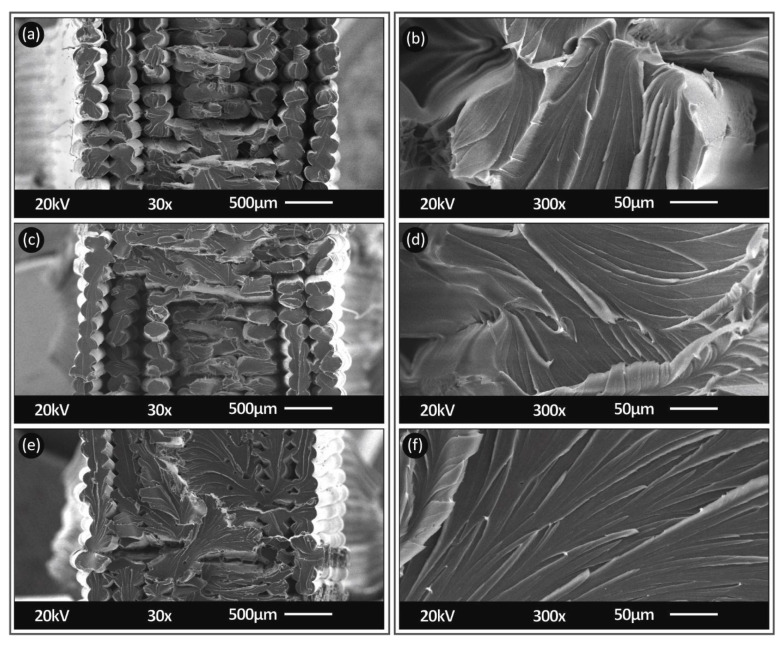
Fracture area SEM captures from PC specimens (built at a 260 °C nozzle temperature, with a 0.2 mm layer thickness) at different elongation speeds of 10 mm/min (**a**,**b**), 100 mm/min (**c**,**d**) and 300 mm/min (**e**,**f**) at 30× (**a**,**c**,**e**) and 300× (**b**,**d**,**f**) magnifications.

**Figure 13:**
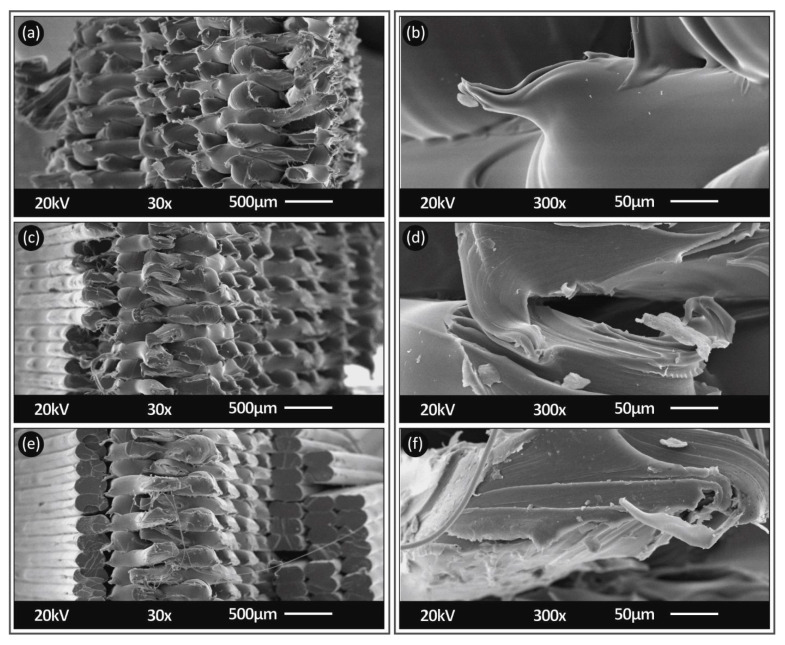
Fracture area SEM captures from TPU specimens (built at a 215 °C nozzle temperature, with a 0.2 mm layer thickness) at different elongation speeds of 10 mm/min (**a**,**b**), 100 mm/min (**c**,**d**) and 300 mm/min (**e**,**f**) at 30× (**a**,**c**,**e**) and 300× (**b**,**d**,**f**) magnifications.

In Figure 11, Figure 12 and Figure 13, SEM images from the side surface and the fracture area of the specimens of this study are shown for both PC and TPU materials. Images were taken from specimens, 3D printed and tested with all the cases studied in this work (nozzle temperature, layer height and elongation speed).

Figure 11 shows, at two different magnifications, an area from the side surface of PC specimens built with a 0.20 mm layer thickness. Layer heights shown are in agreement to the 3D printer settings (0.20 mm) and a consistent inter layer fusion can be observed in the specimens. Similar findings can be observed for TPU material in Figure 11c,d.

Figure 12 shows, at two different magnifications, the fracture area of 3D printed specimens at 260 °C, with a 0.20 mm layer height and tensile tested at 10 mm/min strain rate (Figure 10a,b), 100 mm/min (Figure 10c,d) and 300 mm/min (Figure 10e,f), respectively. For PC material, from the high magnification captures, it can be determined that, increasing the strain rate resulted in a sharper cut for the fracture area (more brittle failure). At the 10 mm/min strain rate, the fracture of the filament strands was rather ductile, and a neck could be observed. As the strain rate increased, the fracture area became more ductile with mixed ductile and brittle areas, while, at the highest tested strain rate of 300 mm/min, the fracture area was entirely brittle. The difference in the calculated tensile strength values for these three cases was less than 5% (in the average values), showing that, although the fracture mechanism on the specimen filament strands changed and strands became more brittle, the increase in the strain rate did not have a significant effect on the overall tensile strength of the specimens. So, the fracture mechanism of the filament strands, in this case, was not the main parameter affecting the fracture of the specimens.

In Figure 13, SEM captures are shown for the same elongation speed for TPU material 3D printed at 215 °C, with a 0.20 mm layer height. TPU brittleness on the fracture area increased with the increase in the elongation speed. In the case of TPU material, this phenomenon was less intense than PC material. At the 10 mm/min strain rate, the fracture of the filament strands was rather ductile, and a neck could be observed. As the strain rate increased, the fracture area became more ductile with mixed ductile and brittle areas. At the highest tested strain rate of 300mm/min, the fracture area was partly ductile and partly brittle, as opposed to PC material, in which the fracture area was entirely brittle, in this case. This was expected, since TPU material has, overall, a more elastic behavior. In this specific case of TPU material, the increase in the strain rate increased the calculated tensile strength values by about 12% (between the lower and the higher strain rate, the difference was calculated in the average values), showing that the change in the fracture mechanism on the specimen filament strands, with the increase in the strain rate, had a more significant effect on the overall tensile strength of the specimens than in PC material.

## 4. Discussion

In the present study, the effects of 3D printer nozzle temperatures and 3D printed specimens layer heights on the tensile strength of PC and TPU materials at various strain rates were investigated. The mechanical response of the materials at different strain rates is a critical parameter related to their behavior in dynamic loading conditions. In parts built with AM technology, this is more critical since the 3D printing build parameters introduce anisotropic behavior and significantly affect the mechanical properties of the 3D printed parts. This is mainly caused due to the achieved quality in the build structure fusion and the intra and inter layer bonding during the building process of the parts [60,61].

**Figure 14:**
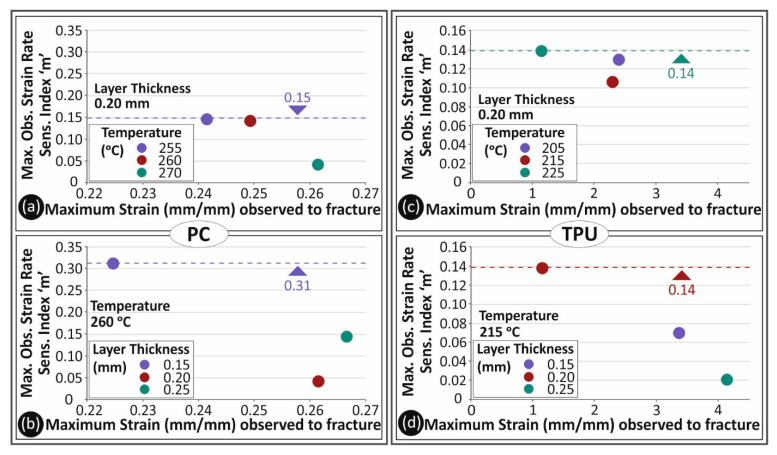
Maximum observed strain rate index ‘*m*’ to maximum strain observed at break (mm/mm) for PC (**a**,**b**) and TPU (**c**,**d**) materials for the different temperatures (**a**,**c**) and the different layer heights (**b**,**d**) studied in this work.

The 3D printing temperature and layer height of the specimens are among the FFF process parameters that significantly affect fusion and bonding of the parts since they affect the flow rates of the materials during the 3D printing process. Changing the temperature parameter exhibited changes in the fusion and intra layer bonding of the PC specimens and a better performance was observed in the tensile tests for specimens built at higher temperatures. Respectively, the strain rate sensitivity decreases with the temperature increase, probably due to stronger bonding of the extruded material. This can be observed in Figure 14a, in which the maximum calculated ‘*m*’ index (describing how sensitive to strain rate a measure is) for 270 °C is far lower than the corresponding value at 260 °C and 255 °C. In Figure 14b, the maximum calculated ‘*m*’ index is shown for the different 3D printing layer heights studied in this work for PC material. In this case, the layer height of 0.20 mm was found to be less prone to changes with the change in the elongation speed. In the literature, layer height decrease usually increases tensile properties [62,63], but this is something that is not absolute for thermoplastic materials. Changing the layer height over or under a specific value may cause difficulties in the extrusion process, mainly due to the thermodynamical flow properties of each material.

For TPU material, Figure 14c,d show the calculated maximum ‘*m*’ index values for the different 3D printing temperatures and layer heights studied herein. Apart from the 225 °C case, which probably caused a change in the internal material structure—specimens 3D printed at this temperature behaved totally different throughout the study—a trend similar to that of PC material was observed for TPU material. Increasing the 3D printing temperature decreased the sensitivity of the materials to the strain rate. The layer height parameter also exhibited the same trend. It is shown, in Figure 14d, that the 0.25 mm layer height exhibited a more stable tensile behavior with the different strain rates studied.

Koomson et al. [64] have conducted a similar research regarding the PC material performance under different strain rates, but not with 3D printed specimens. Results presented in their study show a similar trend to the present study. The brittleness of the specimens increased with the increase in the strain rate, while a test of increasing temperatures exhibited the same trend of stabilization (less sensitivity). Cao et al. [65] and Blumenthal et al. [66] also presented studies with similar results. Results presented in the present study exhibit a greater effect of these than the studies referred to above and this is probably due to the manufacturing process utilized and studied herein (FFF). Miao et al. [67] tested TPU material for a range of strain rates. Their results agree with the results of the present study, also reporting the trend of ‘hardening’ of TPU material with the increase in the strain rate. Generally, the results presented in this study for both materials follow the trends presented in the literature, while the 3D printing parameters tested for their effect on the strain rate provide critical information for the design processes in additive manufacturing applications.

## 5. Conclusions

In this work, the effect of the strain rate on the mechanical response of FFF 3D printed PC and TPU materials was investigated. Strain rate is an important parameter related with dynamic loadings, which are very common in mechanical parts. Although studies have been presented on bulk materials, research on the effect of strain rate on FFF 3D printed parts is very limited and no similar study has been presented so far for the mechanical response of FFF 3D printed specimens with PC and TPU materials. This is important, since 3D printing parameters significantly affect the mechanical properties and add anisotropy to the parts built with this process. For this reason, two of the most critical 3D printing parameters, i.e., layer height and nozzle temperature, affecting the mechanical properties of FFF 3D printed parts were also investigated to evaluate their effect on the mechanical response with the increase in the strain rate. It was found, in all cases studied, that these two 3D printing parameters affect the mechanical response of the two materials more significantly than the strain rate, although, as expected, the strain rate also had an important effect on the mechanical properties of the materials, which is highlighted in the study. For both materials, the tensile strength was more affected than the elastic modulus, with the deviation, for PC material, being 30% between the highest and the lowest values measured, while the maximum difference measured for the elastic modulus was less than 20%. For TPU material, the corresponding deviation was 45% for the tensile strength and 15% for the elastic modulus, with temperature having a more significant effect on this material overall.

The experimental results of PC and TPU specimens are thoroughly analyzed in this study, regarding the effect of nozzle temperature and layer height on the mechanical response of the materials at various strain rates. PC material showed a less sensitive behavior to strain rate for the different parameters studied than TPU material, since, in PC material, the calculated toughness did not intensively change with the change in the different parameters and strain rates. TPU material was more prone to temperature and layer height effect at the various strain rates studied.

Overall, temperature had a more intense effect than layer height. The present study provides all the necessary data for the design of parts for applications exhibiting low strain rate loadings. The optimum 3D printing parameters for nozzle temperature and layer height can be determined for parts built with PC or TPU material with the FFF process, to acquire a rather stable tensile behavior and probably a fractal mechanism able to withstand severe breakdown. In a future work, a neural network, along with a generic algorithm, will be implemented by the authors for the experimental results of this study to optimize the process for the parameters studied herein.

## Figures and Tables

**Table 1:** Fundamental properties of PC and TPU materials used during this study.

Material	PC Emerge 8430-15	TPU Ravathane 140 D70
Property		
Density (g/cm^3^)	1.20	1.25
Tensile stress at break (MPa)	70.0	45.0
Elongation at break (%)	110	350

**Table 2:** Extrusion parameters for PC and TPU materials during filament fabrication in the study.

Material	PC	TPU
Extrusion Parameter		
Heat Zone 1 (°C)	240	205
Heat Zone 2 (°C)	240	205
Heat Zone 3 (°C)	240	205
Heat Zone 4 (°C)	200	185
Screw rotational speed (rpm)	4.8	9.7
Cooling fans (%)	20	40
Winder rotational speed (rpm)	Automatic	Automatic

## Data Availability

The data presented in this study are available on request from the corresponding author.
